# Ethyl 3-(4-methyl­benzyl­idene)carbazate

**DOI:** 10.1107/S1600536810020623

**Published:** 2010-06-05

**Authors:** Yu-Feng Li, Wen-Hai Sheng, Fang-Fang Jian

**Affiliations:** a Microscale Science Institute, Department of Chemistry and Chemical Engineering, Weifang University, Weifang 261061, People’s Republic of China; b Department of Chemistry and Chemical Engineering, Weifang University, Weifang 261061, People’s Republic of China; c Microscale Science Institute, Weifang University, Weifang 261061, People’s Republic of China

## Abstract

There are two mol­ecules in the asymmetric unit of the title compound, C_11_H_14_N_2_O_2_, which have similar conformations. In the crystal, the mol­ecules are linked by N—H⋯O hydrogen bonds, generating *C*(4) chains propagating in [001].

## Related literature

For background to Schiff bases with additional donor groups, see: Borisova *et al.* (2007 ▶); Gradinaru *et al.* (2007 ▶). For a related structure, see: Li *et al.* (2009 ▶).
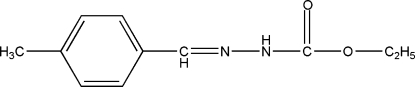


## Experimental

### 

#### Crystal data

C_11_H_14_N_2_O_2_
*M* *_r_* = 206.24
Monoclinic, 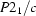

*a* = 15.251 (3) Å
*b* = 8.2853 (17) Å
*c* = 18.139 (4) Å
β = 101.85 (3)°
*V* = 2243.3 (8) Å^3^
*Z* = 8
Mo *K*α radiation
μ = 0.09 mm^−1^
*T* = 293 K
0.22 × 0.21 × 0.20 mm

#### Data collection

Bruker SMART CCD diffractometer
21172 measured reflections
5128 independent reflections
3927 reflections with *I* > 2σ(*I*)
*R*_int_ = 0.037

#### Refinement

*R*[*F* ^2^ > 2σ(*F* ^2^)] = 0.055
*wR*(*F* ^2^) = 0.185
*S* = 1.08
5128 reflections
272 parameters
H-atom parameters constrained
Δρ_max_ = 0.24 e Å^−3^
Δρ_min_ = −0.30 e Å^−3^

### 

Data collection: *SMART* (Bruker, 1997 ▶); cell refinement: *SAINT* (Bruker, 1997 ▶); data reduction: *SAINT*; program(s) used to solve structure: *SHELXS97* (Sheldrick, 2008 ▶); program(s) used to refine structure: *SHELXL97* (Sheldrick, 2008 ▶); molecular graphics: *SHELXTL* (Sheldrick, 2008 ▶); software used to prepare material for publication: *SHELXTL*.

## Supplementary Material

Crystal structure: contains datablocks global, I. DOI: 10.1107/S1600536810020623/hb5473sup1.cif

Structure factors: contains datablocks I. DOI: 10.1107/S1600536810020623/hb5473Isup2.hkl

Additional supplementary materials:  crystallographic information; 3D view; checkCIF report

## Figures and Tables

**Table 1:** Hydrogen-bond geometry (Å, °)

*D*—H⋯*A*	*D*—H	H⋯*A*	*D*⋯*A*	*D*—H⋯*A*
N1*B*—H1*BA*⋯O1*A*	0.86	2.03	2.8747 (17)	165
N1*A*—H1*AA*⋯O1*B*^i^	0.86	2.13	2.9383 (17)	157

Symmetry code: (i) 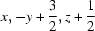.

**Fig. 1.**
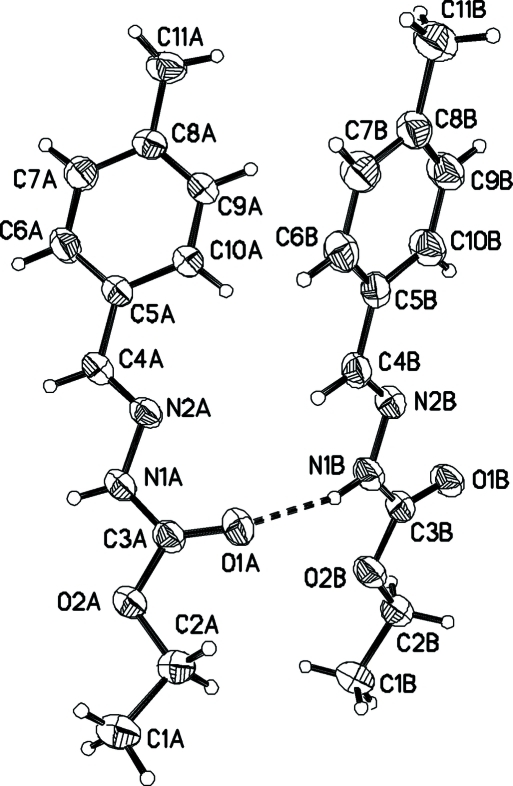
The structure of (I) showing 30% probability displacement ellipsoids. The dashed line indicates a hydrogen bond.

## supplementary crystallographic information

### Comment

Schiff bases bearing additional donor groups represent the important class of heteropolydentate ligands capable of forming mono-, bi-, and polynuclear complexes with metals in coordination chemistry.(Borisova, *et al.*, 2007). Meanwhile, it is an important intermediate compound which have been reported to be compounds with second-order nonlinear optical (NLO) materials (Gradinaru *et al.*, 2007). As part of our search for new schiff base compounds we synthesized the title compound (I), and describe its structure here. The title compound contains two independent molecules in the unit. The dihedral angle between the two independent benzene rings is [72.32 (11)°]. The C1A/C2A/O2A/C3A/O1A/N1A/N2A and C1B/C2B/O2B/C3B/O1B/N1B/N2B planes form dihedral angles of 4.43 (11)° and 2.33 (12)°, respectively, with the benzene planes. In the crystal lattice, the N—H···O intramolecular hydrogen bonds which form chains stable the molecule structures.

Bond lengths and angles are comparable to a related compound (Li *et al.*, 2009).

### Experimental

A mixture of 4-methylbenzaldehyde (0.1 mol), and ethyl carbazate (0.1 mol) was stirred in refluxing ethanol (20 ml) for 4 h to afford the title compound (0.092 mol, yield 92%).  Colourless blocks of (I) were obtained by recrystallization from ethanol at room temperature.

### Refinement

H atoms were fixed geometrically and allowed to ride on their attached atoms, with C—H distances=0.97 Å, and with *U*_iso_=1.2–1.5*U*_eq_.

### Crystal data

**Table:**

C_11_H_14_N_2_O_2_	*F*(000) = 880
*M**_r_* = 206.24	*D*_x_ = 1.221 Mg m^−^^3^
Monoclinic, *P*2_1_/*c*	Mo *K*α radiation, λ = 0.71073 Å
Hall symbol: -P 2ybc	Cell parameters from 3927 reflections
*a* = 15.251 (3) Å	θ = 3.1–27.5°
*b* = 8.2853 (17) Å	µ = 0.09 mm^−^^1^
*c* = 18.139 (4) Å	*T* = 293 K
β = 101.85 (3)°	Block, colorless
*V* = 2243.3 (8) Å^3^	0.22 × 0.21 × 0.20 mm
*Z* = 8	

### Data collection

**Table:**

Bruker SMART CCD diffractometer	3927 reflections with *I* > 2σ(*I*)
Radiation source: fine-focus sealed tube	*R*_int_ = 0.037
graphite	θ_max_ = 27.5°, θ_min_ = 3.1°
ω scans	*h* = −19→19
21172 measured reflections	*k* = −10→10
5128 independent reflections	*l* = −20→23

### Refinement

**Table:**

Refinement on *F*^2^	Secondary atom site location: difference Fourier map
Least-squares matrix: full	Hydrogen site location: inferred from neighbouring sites
*R*[*F*^2^ > 2σ(*F*^2^)] = 0.055	H-atom parameters constrained
*wR*(*F*^2^) = 0.185	*w* = 1/[σ^2^(*F*_o_^2^) + (0.1115*P*)^2^ + 0.1779*P*] where *P* = (*F*_o_^2^ + 2*F*_c_^2^)/3
*S* = 1.08	(Δ/σ)_max_ < 0.001
5128 reflections	Δρ_max_ = 0.24 e Å^−^^3^
272 parameters	Δρ_min_ = −0.30 e Å^−^^3^
0 restraints	Extinction correction: *SHELXL97* (Sheldrick, 2008), Fc^*^=kFc[1+0.001xFc^2^λ^3^/sin(2θ)]^-1/4^
Primary atom site location: structure-invariant direct methods	Extinction coefficient: 0.064 (6)

### Special details

**Table:**

Geometry. All e.s.d.'s (except the e.s.d. in the dihedral angle between two l.s. planes) are estimated using the full covariance matrix. The cell e.s.d.'s are taken into account individually in the estimation of e.s.d.'s in distances, angles and torsion angles; correlations between e.s.d.'s in cell parameters are only used when they are defined by crystal symmetry. An approximate (isotropic) treatment of cell e.s.d.'s is used for estimating e.s.d.'s involving l.s. planes.
Refinement. Refinement of *F*^2^ against ALL reflections. The weighted *R*-factor *wR* and goodness of fit *S* are based on *F*^2^, conventional *R*-factors *R* are based on *F*, with *F* set to zero for negative *F*^2^. The threshold expression of *F*^2^ > σ(*F*^2^) is used only for calculating *R*-factors(gt) *etc*. and is not relevant to the choice of reflections for refinement. *R*-factors based on *F*^2^ are statistically about twice as large as those based on *F*, and *R*- factors based on ALL data will be even larger.

### Fractional atomic coordinates and isotropic or equivalent isotropic displacement parameters (Å^2^)

**Table:**

	*x*	*y*	*z*	*U*_iso_*/*U*_eq_	
N2A	0.37582 (8)	0.81674 (15)	0.31081 (7)	0.0581 (3)	
O2A	0.59141 (7)	0.95340 (14)	0.38400 (6)	0.0669 (3)	
O1B	0.46162 (7)	0.81088 (16)	0.00172 (6)	0.0724 (4)	
N1A	0.46022 (8)	0.83572 (16)	0.35399 (7)	0.0617 (3)	
H1AA	0.4760	0.7844	0.3958	0.074*	
O2B	0.57247 (7)	0.80498 (16)	0.10534 (6)	0.0720 (3)	
N1B	0.44923 (9)	0.93461 (17)	0.11143 (6)	0.0642 (4)	
H1BA	0.4757	0.9577	0.1568	0.077*	
N2B	0.36356 (8)	0.98639 (16)	0.08381 (6)	0.0607 (3)	
C5A	0.23044 (9)	0.70401 (17)	0.29857 (8)	0.0542 (3)	
O1A	0.50636 (8)	1.00212 (17)	0.26947 (6)	0.0762 (4)	
C3A	0.51804 (10)	0.93606 (19)	0.32980 (7)	0.0570 (3)	
C5B	0.23472 (10)	1.12384 (18)	0.10486 (7)	0.0574 (4)	
C3B	0.49157 (10)	0.84683 (19)	0.06658 (8)	0.0602 (4)	
C8A	0.04993 (10)	0.66046 (18)	0.22712 (9)	0.0599 (4)	
C4A	0.32349 (10)	0.72637 (19)	0.33783 (8)	0.0598 (4)	
H4AA	0.3444	0.6734	0.3832	0.072*	
C4B	0.32641 (11)	1.06587 (18)	0.12903 (8)	0.0598 (4)	
H4BA	0.3578	1.0871	0.1777	0.072*	
C9A	0.10661 (10)	0.7566 (2)	0.19601 (8)	0.0646 (4)	
H9AA	0.0845	0.8079	0.1504	0.077*	
C10A	0.19508 (10)	0.77894 (19)	0.23044 (8)	0.0613 (4)	
H10A	0.2314	0.8447	0.2079	0.074*	
C6B	0.19536 (12)	1.2192 (2)	0.15151 (9)	0.0701 (4)	
H6BA	0.2277	1.2459	0.1992	0.084*	
C6A	0.17352 (12)	0.6081 (2)	0.32983 (9)	0.0718 (4)	
H6AA	0.1954	0.5560	0.3753	0.086*	
C2A	0.65982 (10)	1.0586 (2)	0.36603 (10)	0.0683 (4)	
H2AB	0.6337	1.1604	0.3461	0.082*	
H2AC	0.6883	1.0087	0.3286	0.082*	
C10B	0.18358 (12)	1.0863 (2)	0.03459 (9)	0.0721 (4)	
H10B	0.2083	1.0225	0.0019	0.087*	
C8B	0.05775 (12)	1.2381 (2)	0.05846 (10)	0.0684 (4)	
C9B	0.09748 (13)	1.1412 (2)	0.01226 (10)	0.0763 (5)	
H9BA	0.0649	1.1128	−0.0351	0.092*	
C2B	0.62646 (11)	0.7096 (3)	0.06543 (10)	0.0753 (5)	
H2BB	0.6391	0.7694	0.0228	0.090*	
H2BC	0.5954	0.6108	0.0470	0.090*	
C7A	0.08469 (12)	0.5880 (2)	0.29489 (10)	0.0742 (5)	
H7AA	0.0478	0.5240	0.3177	0.089*	
C11A	−0.04662 (11)	0.6351 (3)	0.18905 (11)	0.0790 (5)	
H11A	−0.0593	0.6943	0.1426	0.118*	
H11B	−0.0848	0.6726	0.2214	0.118*	
H11C	−0.0572	0.5222	0.1789	0.118*	
C1B	0.71149 (13)	0.6717 (3)	0.12010 (12)	0.0866 (6)	
H1BB	0.7493	0.6069	0.0957	0.130*	
H1BC	0.6979	0.6137	0.1622	0.130*	
H1BD	0.7418	0.7704	0.1375	0.130*	
C11B	−0.03689 (13)	1.2974 (3)	0.03316 (13)	0.0880 (6)	
H11D	−0.0531	1.3622	0.0722	0.132*	
H11E	−0.0768	1.2068	0.0228	0.132*	
H11F	−0.0413	1.3611	−0.0117	0.132*	
C7B	0.10868 (13)	1.2758 (2)	0.12853 (10)	0.0751 (5)	
H7BA	0.0841	1.3406	0.1609	0.090*	
C1A	0.72665 (12)	1.0856 (3)	0.43748 (11)	0.0850 (5)	
H1AB	0.7731	1.1560	0.4280	0.127*	
H1AC	0.7523	0.9842	0.4563	0.127*	
H1AD	0.6975	1.1342	0.4741	0.127*	

### Atomic displacement parameters (Å^2^)

**Table:**

	*U*^11^	*U*^22^	*U*^33^	*U*^12^	*U*^13^	*U*^23^
N2A	0.0505 (6)	0.0690 (8)	0.0487 (6)	0.0009 (5)	−0.0044 (5)	−0.0021 (5)
O2A	0.0542 (6)	0.0850 (7)	0.0551 (6)	−0.0096 (5)	−0.0035 (5)	0.0072 (5)
O1B	0.0614 (6)	0.1030 (9)	0.0472 (6)	0.0021 (6)	−0.0020 (5)	−0.0095 (5)
N1A	0.0536 (6)	0.0772 (8)	0.0471 (6)	−0.0042 (6)	−0.0061 (5)	0.0053 (5)
O2B	0.0601 (6)	0.1023 (9)	0.0481 (5)	0.0065 (6)	−0.0017 (5)	−0.0001 (5)
N1B	0.0608 (7)	0.0869 (9)	0.0402 (6)	0.0023 (6)	−0.0008 (5)	−0.0012 (5)
N2B	0.0597 (7)	0.0715 (8)	0.0474 (6)	−0.0014 (6)	0.0031 (5)	0.0050 (5)
C5A	0.0523 (7)	0.0578 (8)	0.0488 (7)	0.0031 (5)	0.0018 (6)	−0.0010 (5)
O1A	0.0757 (7)	0.1016 (9)	0.0463 (6)	−0.0109 (6)	0.0007 (5)	0.0080 (5)
C3A	0.0547 (7)	0.0680 (9)	0.0450 (7)	0.0018 (6)	0.0023 (6)	−0.0040 (6)
C5B	0.0662 (8)	0.0594 (8)	0.0453 (7)	−0.0043 (6)	0.0082 (6)	0.0064 (6)
C3B	0.0567 (8)	0.0755 (9)	0.0442 (7)	−0.0056 (6)	0.0007 (6)	0.0042 (6)
C8A	0.0523 (7)	0.0629 (8)	0.0612 (8)	0.0035 (6)	0.0042 (6)	−0.0077 (6)
C4A	0.0572 (8)	0.0666 (9)	0.0496 (7)	0.0027 (6)	−0.0034 (6)	0.0031 (6)
C4B	0.0657 (8)	0.0679 (9)	0.0431 (6)	−0.0045 (7)	0.0051 (6)	0.0040 (6)
C9A	0.0560 (8)	0.0822 (10)	0.0514 (7)	0.0064 (7)	0.0014 (6)	0.0093 (7)
C10A	0.0529 (7)	0.0740 (9)	0.0550 (8)	0.0014 (6)	0.0061 (6)	0.0110 (7)
C6B	0.0803 (10)	0.0777 (10)	0.0496 (8)	0.0010 (8)	0.0072 (7)	−0.0044 (7)
C6A	0.0706 (9)	0.0798 (11)	0.0590 (8)	−0.0036 (8)	−0.0009 (7)	0.0188 (7)
C2A	0.0554 (8)	0.0760 (10)	0.0712 (9)	−0.0050 (7)	0.0075 (7)	0.0037 (8)
C10B	0.0715 (10)	0.0894 (12)	0.0523 (8)	0.0067 (8)	0.0053 (7)	−0.0092 (7)
C8B	0.0705 (9)	0.0667 (9)	0.0666 (9)	0.0018 (7)	0.0104 (8)	0.0097 (7)
C9B	0.0732 (10)	0.0930 (12)	0.0564 (8)	0.0049 (9)	−0.0014 (8)	−0.0046 (8)
C2B	0.0592 (9)	0.1050 (13)	0.0598 (9)	0.0058 (8)	0.0076 (7)	0.0037 (8)
C7A	0.0643 (9)	0.0819 (11)	0.0731 (10)	−0.0112 (8)	0.0068 (8)	0.0157 (8)
C11A	0.0554 (8)	0.0934 (13)	0.0820 (11)	−0.0039 (8)	0.0001 (8)	−0.0089 (9)
C1B	0.0635 (10)	0.1040 (14)	0.0834 (12)	0.0040 (9)	−0.0052 (9)	0.0051 (10)
C11B	0.0766 (12)	0.0861 (13)	0.0963 (14)	0.0124 (9)	0.0058 (10)	0.0095 (10)
C7B	0.0807 (11)	0.0774 (11)	0.0670 (10)	0.0106 (8)	0.0146 (9)	−0.0050 (8)
C1A	0.0609 (9)	0.1028 (14)	0.0836 (12)	−0.0139 (9)	−0.0028 (9)	−0.0013 (10)

### Geometric parameters (Å, °)

**Table:**

N2A—C4A	1.2641 (19)	C6B—H6BA	0.9300
N2A—N1A	1.3716 (17)	C6A—C7A	1.383 (2)
O2A—C3A	1.3371 (17)	C6A—H6AA	0.9300
O2A—C2A	1.4471 (19)	C2A—C1A	1.492 (2)
O1B—C3B	1.2086 (18)	C2A—H2AB	0.9700
N1A—C3A	1.349 (2)	C2A—H2AC	0.9700
N1A—H1AA	0.8600	C10B—C9B	1.370 (3)
O2B—C3B	1.3345 (18)	C10B—H10B	0.9300
O2B—C2B	1.439 (2)	C8B—C7B	1.382 (3)
N1B—C3B	1.350 (2)	C8B—C9B	1.386 (3)
N1B—N2B	1.3687 (18)	C8B—C11B	1.505 (3)
N1B—H1BA	0.8600	C9B—H9BA	0.9300
N2B—C4B	1.2722 (19)	C2B—C1B	1.495 (2)
C5A—C6A	1.382 (2)	C2B—H2BB	0.9700
C5A—C10A	1.389 (2)	C2B—H2BC	0.9700
C5A—C4A	1.4631 (19)	C7A—H7AA	0.9300
O1A—C3A	1.2036 (17)	C11A—H11A	0.9600
C5B—C6B	1.382 (2)	C11A—H11B	0.9600
C5B—C10B	1.386 (2)	C11A—H11C	0.9600
C5B—C4B	1.458 (2)	C1B—H1BB	0.9600
C8A—C7A	1.373 (2)	C1B—H1BC	0.9600
C8A—C9A	1.379 (2)	C1B—H1BD	0.9600
C8A—C11A	1.507 (2)	C11B—H11D	0.9600
C4A—H4AA	0.9300	C11B—H11E	0.9600
C4B—H4BA	0.9300	C11B—H11F	0.9600
C9A—C10A	1.378 (2)	C7B—H7BA	0.9300
C9A—H9AA	0.9300	C1A—H1AB	0.9600
C10A—H10A	0.9300	C1A—H1AC	0.9600
C6B—C7B	1.384 (2)	C1A—H1AD	0.9600
			
C4A—N2A—N1A	116.09 (12)	C1A—C2A—H2AC	110.3
C3A—O2A—C2A	115.62 (12)	H2AB—C2A—H2AC	108.6
C3A—N1A—N2A	118.98 (12)	C9B—C10B—C5B	121.43 (16)
C3A—N1A—H1AA	120.5	C9B—C10B—H10B	119.3
N2A—N1A—H1AA	120.5	C5B—C10B—H10B	119.3
C3B—O2B—C2B	116.12 (12)	C7B—C8B—C9B	116.98 (16)
C3B—N1B—N2B	119.14 (12)	C7B—C8B—C11B	121.96 (17)
C3B—N1B—H1BA	120.4	C9B—C8B—C11B	121.05 (16)
N2B—N1B—H1BA	120.4	C10B—C9B—C8B	121.66 (16)
C4B—N2B—N1B	116.38 (12)	C10B—C9B—H9BA	119.2
C6A—C5A—C10A	117.40 (13)	C8B—C9B—H9BA	119.2
C6A—C5A—C4A	120.10 (13)	O2B—C2B—C1B	106.82 (15)
C10A—C5A—C4A	122.49 (13)	O2B—C2B—H2BB	110.4
O1A—C3A—O2A	124.40 (14)	C1B—C2B—H2BB	110.4
O1A—C3A—N1A	126.25 (14)	O2B—C2B—H2BC	110.4
O2A—C3A—N1A	109.35 (12)	C1B—C2B—H2BC	110.4
C6B—C5B—C10B	117.28 (15)	H2BB—C2B—H2BC	108.6
C6B—C5B—C4B	120.97 (13)	C8A—C7A—C6A	121.28 (15)
C10B—C5B—C4B	121.75 (15)	C8A—C7A—H7AA	119.4
O1B—C3B—O2B	124.93 (15)	C6A—C7A—H7AA	119.4
O1B—C3B—N1B	125.97 (15)	C8A—C11A—H11A	109.5
O2B—C3B—N1B	109.10 (12)	C8A—C11A—H11B	109.5
C7A—C8A—C9A	117.39 (14)	H11A—C11A—H11B	109.5
C7A—C8A—C11A	120.69 (15)	C8A—C11A—H11C	109.5
C9A—C8A—C11A	121.92 (15)	H11A—C11A—H11C	109.5
N2A—C4A—C5A	120.84 (13)	H11B—C11A—H11C	109.5
N2A—C4A—H4AA	119.6	C2B—C1B—H1BB	109.5
C5A—C4A—H4AA	119.6	C2B—C1B—H1BC	109.5
N2B—C4B—C5B	120.22 (13)	H1BB—C1B—H1BC	109.5
N2B—C4B—H4BA	119.9	C2B—C1B—H1BD	109.5
C5B—C4B—H4BA	119.9	H1BB—C1B—H1BD	109.5
C10A—C9A—C8A	122.00 (14)	H1BC—C1B—H1BD	109.5
C10A—C9A—H9AA	119.0	C8B—C11B—H11D	109.5
C8A—C9A—H9AA	119.0	C8B—C11B—H11E	109.5
C9A—C10A—C5A	120.56 (14)	H11D—C11B—H11E	109.5
C9A—C10A—H10A	119.7	C8B—C11B—H11F	109.5
C5A—C10A—H10A	119.7	H11D—C11B—H11F	109.5
C5B—C6B—C7B	121.16 (15)	H11E—C11B—H11F	109.5
C5B—C6B—H6BA	119.4	C8B—C7B—C6B	121.48 (16)
C7B—C6B—H6BA	119.4	C8B—C7B—H7BA	119.3
C5A—C6A—C7A	121.36 (14)	C6B—C7B—H7BA	119.3
C5A—C6A—H6AA	119.3	C2A—C1A—H1AB	109.5
C7A—C6A—H6AA	119.3	C2A—C1A—H1AC	109.5
O2A—C2A—C1A	106.88 (14)	H1AB—C1A—H1AC	109.5
O2A—C2A—H2AB	110.3	C2A—C1A—H1AD	109.5
C1A—C2A—H2AB	110.3	H1AB—C1A—H1AD	109.5
O2A—C2A—H2AC	110.3	H1AC—C1A—H1AD	109.5

### Hydrogen-bond geometry (Å, °)

**Table:**

*D*—H···*A*	*D*—H	H···*A*	*D*···*A*	*D*—H···*A*
N1B—H1BA···O1A	0.86	2.03	2.8747 (17)	165
N1A—H1AA···O1B^i^	0.86	2.13	2.9383 (17)	157

Symmetry codes: (i) *x*, −*y*+3/2, *z*+1/2.
